# Increasing the Reliability and Versatility of Jellyfish Biohybrid Vehicles via Species Selection and Rhopalia Removal

**DOI:** 10.3390/biomimetics10120810

**Published:** 2025-12-03

**Authors:** Simon R. Anuszczyk, Noa Yoder, John H. Costello, John O. Dabiri, Brad J. Gemmell, Kelsi M. Rutledge, Sean P. Colin

**Affiliations:** 1 Graduate Aerospace Laboratories and Department of Mechanical and Civil Engineering, California Institute of Technology, Pasadena, CA 91125, USA; 2 Department of Biology, Providence College, Providence, RI 02918, USA; 3 Whitman Center, Marine Biological Laboratory, Woods Hole, MA 02543, USA; 4 Department of Integrative Biology, University of South Florida, Tampa, FL 33620, USA; 5 Sensors and Sonar Division, Naval Undersea Warfare Center, Newport, RI 02841, USA; 6 Department of Marine Biology and Environmental Science, Roger Williams University, Bristol, RI 02809, USA

**Keywords:** cyborg jellyfish, robotic vehicle, ocean sampling, swimming kinematics, medusa, box jellyfish

## Abstract

Jellyfish biohybrid robots have been demonstrated to be successfully programmed to perform vertical sampling profiles of the ocean water column. However, the jellyfish’s endogenous swimming behavior can interfere with the controlled swim cycles, decreasing performance. Further, the model animal used to date, *Aurelia aurita*, is a relatively slow, weakly swimming species. To enhance the performance of the biohybrid vehicles, we tested whether removing the swimming pacemaker of the jellyfish, the rhopalia, eliminated endogenous movements and enhanced responsiveness of the jellyfish to the swim controller. Further, we tested the responsiveness of two fast-swimming jellyfish species, the rhizostome *Cassiopea* spp. and the cubomedusae *Alatina alata*. We found in field trials, where the jellyfish swam controlled vertical profiles in the ocean, that removal of rhopalia eliminated all endogenous behaviors and greatly improved the responsiveness of the jellyfish to the swim controller. This was especially true for species with strong endogenous behaviors that prevented the controller from manipulating swim pulses. Further, we found that both *Cassiopea* spp. and *A. alata* were highly responsive to the swim controller and that these faster-swimming jellyfish species greatly increased the speed at which the biohybrid vehicle could traverse vertical profiles in the water column. These enhancements greatly increase the reliability and versatility of jellyfish biohybrid robot vehicles.

## 1. Introduction

Ocean monitoring and observation is central to understanding our impact on climatic and biological systems. Models used to predict how oceans may impact, and are impacted by, climate and how the use of ocean resources, such as fish and minerals, affect ocean ecosystems are only as reliable as the data they are based on [1]. However, ocean observation is most often limited by access to remote habitats and the funding needed to deploy sensors [2]. Autonomous vehicles (AUVs) equipped with sensors are often seen as the best solution to ocean observation challenges. AUVs enable access to remote habitats and can be deployed for extended sampling missions [2,3,4]. However, they are too expensive to scale to the full ocean and the data they can collect is often limited by battery power and the habitats that they can operate in [5,6].

Biohybrid robots are robotic systems that incorporate biological components or control entire organisms to serve as autonomous vehicles. Integrating biological organisms into robotics has been seen as a solution to many of the biggest challenges in AUV robotics, including power consumption, cost and adaptability to complex habitats [7]. Within the family of biohybrid robots are those which robotically manipulate an entire animal and rely on the animal’s natural propulsion to transport the vehicle. As potential low-cost options, biohybrid vehicles equipped with sensors may facilitate ocean observation by enabling researchers to deploy large numbers of vehicles throughout different habitats. And because they are made primarily from living material, they can biodegrade, limiting their impact on ecosystems [8].

There have only been a few successful attempts at biohybrid robots based on whole-animal manipulation. Goldfish and lamprey have been stimulated using electrical or optogenetic stimulation, respectively, to control the movements of their fins [9,10,11]. Using electrical stimulation along the lateral line, eels have been controlled to perform short bouts of forward and reverse swimming at increased swimming speeds [12]. However, fish have complex neuro-muscular systems that are difficult to control, making it difficult to override the fish’s natural behavior. Further, habituation to the stimulus can decrease the responsiveness of the fish. Jellyfish have also been manipulated to function as biohybrid vehicles [13,14,15,16]. Having much simpler neuro-muscular systems, jellyfish can be electronically stimulated to swim for long durations at enhanced swimming speeds without sustaining damage [15,16]. Jellyfish also require the lowest level of electrical power per unit mass to control their swimming [13]. However, similarly to biohybrid fish, the natural swimming behavior of jellyfish interferes with the stimulated bell contractions, which can reduce control and performance of the biohybrid jellyfish [13].

The scyphozoan jellyfish *Aurelia aurita* is the only jellyfish species that has been used as a biohybrid robot platform. *A. aurita* and other scyphozoans use rowing propulsion to swim, and their propulsion has been shown to be the most efficient mode of swimming among all animals [17,18,19]. *A. aurita*, and other scyphozoans, swim by rhythmically contracting and relaxing their mesoglea bells to generate large vortex rings in their wake. The bell contractions are initiated by rhopalial pacemakers that are distributed around the bell margin of the jellyfish [20]. Jellyfish typically have about 8 rhopalia but some species can have 16 or more. To initiate a contraction, a rhopalium sends an electrical impulse that is conducted to the other pacemakers through the nerve-net. This suppresses the other rhopalia and stimulates the jellyfish muscles to contract the bell [20]. During contraction, collagen fibers in the mesoglea are stretched. After contraction, the muscles relax and, due to relaxation of the elastic strain stored in mesogleal collogen fibers, the bell recoils to its resting size and shape. The high swimming efficiency and relatively simple neuro-muscular system that controls swimming make scyphozoan jellyfish ideal animal models for biohybrid vehicles [13]. However, compared to other scyphozoan species, *A. aurita* is a slow, weak swimmer, making it a less than optimal animal model for applications that require faster-moving biohybrid robots.

The basic circuitry of the scyphozoan neural system allows jellyfish to be stimulated to swim by activating an electrode that has been inserted into their sub-umbrellar surface. A 5 mV electrical impulse is sufficient to initiate bell contraction; however, unlike the natural signals from the rhopalia, the external electrical impulse does not suppress the rhopalia from also initiating pulses [13]. The endogenous bell contractions initiated by the rhopalia interfere with externally stimulated bell contractions and can affect swimming performance. In addition, scyphozoan jellyfish swim nearly continuously, which can prevent the biohybrid robot from stopping or maneuvering on command. These endogenous behaviors have been cited as some of the major challenges with jellyfish biohybrid robots. In fact, endogenous animal behaviors have been cited as one of the main obstacles to whole-animal biohybrid robots [7].

**Figure 1:**
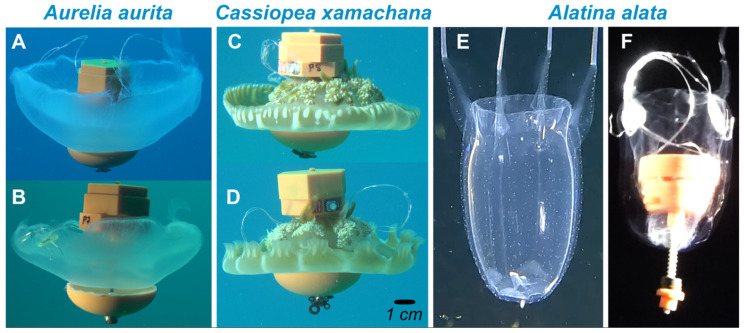
Jellyfish species used as biohybrid robots swimming in the natural water column. (**A**) Non-excised and (**B**) excised *Aurelia aurita*. (**C**) Non-excised and (**D**) excised *Cassiopea xamachana*. (**E**) Cubomedusa *Alatina alata* without swim controller. (**F**) *A. alata* with swim controller.

The goal of this study was to advance jellyfish biohybrid vehicle design by enhancing the control and speed of biohybrid vehicles. While other studies have improved the engineering to increase the reliability and versatility of jellyfish biohybrid robots—e.g., improving the swim controller, the connection to the jellyfish and the ballasting of the controller payload—no other studies to date have tried to leverage jellyfish biology to enhance the performance of these robot systems. In this study, we evaluated how leveraging two biological traits—the natural rhopalia pacemakers and the natural swimming performance of different species—can improve the reliability and versatility of jellyfish biohybrid robots. Using biological traits to improve biohybrid robots takes advantage of the evolutionary traits of these animals that have already optimized animal performance. To enhance the control of swimming, we tested whether removing the rhopalial pacemakers around the bell margin eliminated endogenous bell contractions of the jellyfish (Supplementary Figure S1). To enhance the speed of biohybrid vehicles, we tested the ability to control the swimming of two different fast-swimming jellyfish species. First, we tested another scyphozoan jellyfish, *Cassiopea* spp. (Figure 1C,D). *Cassiopea* is a rhizostome, which are typically stronger, faster swimmers than semaeostomes like *A. aurita* (Figure 1A,B). We also tested a cubomedusae, *Alatina alata*, which uses jet propulsion to swim instead of the rowing propulsion described above that is used by scyphozoan jellyfish (Figure 1E,F). Specifically, we compared the responsiveness and swimming speed of *Cassiopea* spp. and *A. alata* to that of *A. aurita* by using a swim controller to stimulate them to swim across 6 m vertical transects in natural ocean waters. For *Cassiopea* spp. and *A. aurita*, we also conducted experiments comparing the responsiveness of animals with intact rhopalia to that of animals whose rhopalia were all removed to determine if the jellyfish would be equally responsive to the swim controller if we remove the endogenous swimming of the jellyfish.

## 2. Materials and Methods

### 2.1. Animals and Care

In situ experiments were performed with the scyphozoan jellyfish *Cassiopea xamachana* and *Aurelia aurita* offshore from Long Key, FL, and with the cubomedusa *Alatina alata* offshore from Kona, HI. *C. xamachana* individuals were collected by hand from the waters adjacent to the Keys Marine Laboratory (KML), Long Key, FL, USA, and *A. aurita* individuals were supplied by the Cabrillo Aquarium and Aquarium of the Pacific and shipped to the KML. Medusae were kept in large (>100 L) tanks supplied with filtered seawater at ambient temperature (same temperature as the dive site) and fed natural plankton. All experiments were conducted within 3 days of animals being collected or arriving at the lab.

### 2.2. Biohybrid Robotic System

We modified the robotic system described in for our field experiments. The swim controller comprised a mini processor (TinyLily, TinyCircuits, Akron, OH, USA) and a 10 mAh lithium polymer cell (GM201212, PowerStream Technology Inc., Orem, UT, USA) in plastic housing made entirely from polypropylene pieces sealed with hot-melt adhesives. The housing was ballasted with stainless steel washers to keep the system neutrally buoyant in seawater. Two electrodes were assembled using perfluoroalkoxy-coated silver wires and platinum rod tips (A-M Systems, Sequim, WA, USA) connected in series to red-light-emitting diodes (LEDs); (TinyLily 0402, TinyCircuits, Akron, OH, USA) they functioned as a visualization tool to identify when impulses were sent to the jellyfish (Supplementary Figure S1). Platinum wire tips were hooked to the subumbrellar surface of the jellyfish to improve attachment. The robotic system was attached to the jellyfish bell in three locations: a plastic threaded pin connected to the housing was inserted into the center of the manubrium from the subumbrellar surface, and 2 electrodes were inserted into the subumbrellar tissue. The other end of the plastic threaded pin was attached to a plastic forebody, similarly to the method of [16].

### 2.3. Field Experiments

To examine the effects of jellyfish species type on the responsiveness and swimming speeds of biohybrid robots, we conducted field experiments where the different robot treatments swam from the surface down to a 6 m depth. Five different treatments were compared (*C. xamachana* with and without rhopalia, *A. aurita* with and without rhopalia, *A. alata* with rhopalia), with 2 replicate individuals used for each treatment. For the experiments, the swim controllers were programmed to start sending impulses at 0.5 Hz at the surface and to stop sending impulses at a 6 m depth. All the robot treatments were neutrally buoyant. SCUBA divers videoed descending jellyfish at 30 fps using SONY camcorders (SONY AX700,New York, NY, USA) in Gates underwater housings (Gates Inc, Poway, CA, USA). The red LED signals on the swim controller were visible in the video recordings and indicated when electrical impulses were sent.

Rhopalia were excised using surgical scissors by cutting small v-shaped grooves into the bell margin (Supplementary Figure S1). The jellyfish were unharmed by the procedure and were able to be maintained in good condition; in fact, rhopalia are known to be able to be regenerated by scyphozoan and cubozoan jellyfish within 2 weeks. All the experiments were conducted within 2 days of the rhopalial excision to ensure the pacemakers had not regenerated.

### 2.4. Laboratory Experiments

In the laboratory, we performed additional tests to evaluate the relationship between stimulation frequency and jellyfish pulse frequency. These experiments were conducted in Kona, HI, with *A. alata* and *Cassiopea andromeda*. *C. andromeda* were supplied by the Waikiki Aquarium. For these experiments individual jellyfish were placed into tanks with the swim controller connected to their bells. They were video-recorded using a GoPro camera (GoPro Inc., San Mateo, CA, USA) at 30 fps (Supplementary Figure S2). The swim controller was set to different frequencies ranging from 0.5 Hz to 2.5 Hz.

### 2.5. Data and Statistical Analysis

Videos of descending biohybrid jellyfish were analyzed to quantify the correspondence between electrical impulses and bell contractions. The timing of electrical impulses was identified by the flashing of the red LED attached to the inserted electrode. The bell contractions were quantified by measuring the bell diameter and height and calculating the fineness ratio (height/diameter). The videos were scaled by measuring the diameter of the swim controller, which was known to be 4 cm in diameter. This enabled us to ensure that the diameter did not change with changes in the distance the animal was from the camera. Minimum and maximum bell contraction correspond to minimum and maximum fineness ratios, respectively. Both field and laboratory videos were analyzed by hand to determine the pulse frequencies of the jellyfish. Pulse frequencies were measured manually where contractions were noted to start at the first frame when the bell diameter began to decrease. From this analysis we calculated the time delay between the impulse and the onset of bell contraction, the contraction frequency and the impulse frequency (which was set to 0.5 Hz).

Descent speed was measured by changes in pressure over time, quantified by pressure sensors in the swim control unit. To quantify an average descent speed, the depth of the jellyfish was plotted versus time, and the slope of that line was used to estimate an average descent rate.

## 3. Results

### 3.1. Control of Bell Contraction for Excised Versus Non-Excised Jellyfish

**Figure 2:**
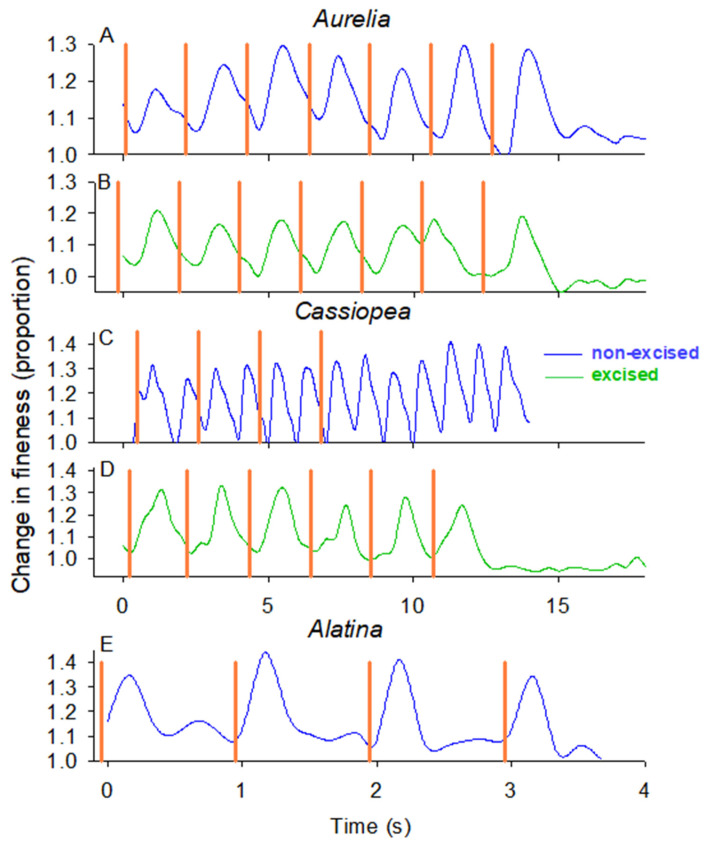
Timing of bell contractions versus stimulation signals from the swim controller for individual (**A**) *Aurelia* non-excised, (**B**) *Aurelia* excised, (**C**) *Cassiopea* non-excised, (**D**) *Cassiopea* excised and (**E**) *Alatina* non-excised. The fineness ratio (or aspect ratio) illustrates when the jellyfish bells are in their completely relaxed, expanded state (minimum fineness ratio) or in their contracted state (maximum fineness ratio). A swim cycle begins when the jellyfish in the relaxed state initiates muscle contractions that cause the bell to contract, thus increasing the fineness ratio. The orange vertical lines indicate the timing of the stimulation from the swim controller.

**Figure 3:**
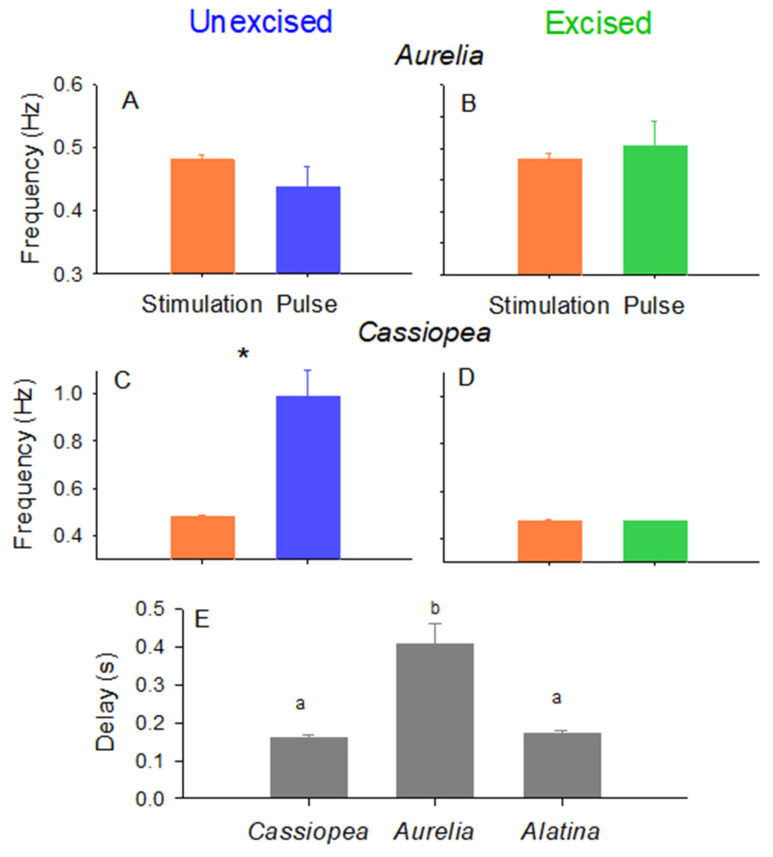
Comparison of stimulation frequency (orange) to jellyfish pulse frequency (blue for non-excised and green for excised) and delay (grey) for (**A**) *Aurelia* non-excised, (**B**) *Aurelia* excised, (**C**) *Cassiopea* non-excised and (**D**) *Cassiopea* excised. When the swim controller was able to manipulate pulses, the stimulation and pulse frequencies were the same. The asterisk indicates when the stimulation and the pulse frequencies were significantly different (*T*-test, *p* < 0.05). (**E**) The time delay between when the jellyfish received the stimulation and the bell began to contract to start a swim cycle. Species with the same letters were not significantly different from each other, while species with different delay times are indicated by different letters (Student–Newman–Keuls pair-wise comparison, *p* < 0.05).

In the field, we evaluated the ability of the swim controller unit to control bell contractions by comparing the timing of bell contractions of *Cassiopea xamachana* and *Aurelia aurita*, with and without rhopalia, to the timing of the electrical impulses from the control unit. Figure 2 visualizes the timing of the stimulation impulse (orange lines) and the timing of the bell contractions (changes in fineness ratio where peak fineness occurs at peak contraction and minimum fineness at bell relaxation). The controller reliably initiated bell contractions for both non-excised and excised *A. aurita* (Figure 2A,B; Supplementary Figure S3) where the pulsation frequency was the same as the stimulation frequency (Figure 3A,B). In contrast, the bell contractions of the non-excised *C. xamachana* (with rhopalia pacemakers) did not correspond with the impulse signals from the controller (Figure 2C). In fact, the non-excised *C. xamachana* swam with a contraction frequency that was twice the frequency of the electrical impulses (Figure 3C), and after the electrical impulses stopped, the medusae continued pulsing at that same inherent frequency. In contrast, the excised *C. xamachana* contracted their bells synchronously with the electrical impulse at a frequency equal to the electrical impulse at 0.47 Hz (Figure 2D and Figure 3B and Supplementary Figure S4; *T*-test, *p* > 0.05). Further, as soon as the electrical impulse stopped, the medusae stopped contracting their bells. Of note, the excised bells of the two species reacted to the stimulus at different speeds, where *C. xamachana* initiated bell contractions in less than half the time of *A. aurita*, contracting 0.16 s after the stimulus vs. 0.41 s for *A. aurita* (Figure 3E).

### 3.2. Control of Cubomedusa Alatina Alata

We were able to collect two individuals of *Alatina alata*, an oceanic epipelagic cubomedusae, and evaluate if this class of medusae responds to the stimulus as well as scyphozoan medusae. We found that the controller also reliably initiated bell contractions for excised *A. alata* (Figure 2E and Supplementary Figure S5), with the bell contracting at the same frequency as the stimulus (*T*-test, *p* > 0.05). In fact, the bell contractions of *A. alata* were initiated just as quickly as those of *C. xamachana* after the stimulus, contracting 0.17 s after the stimulus.

### 3.3. Comparison of Descent Rates

**Figure 4:**
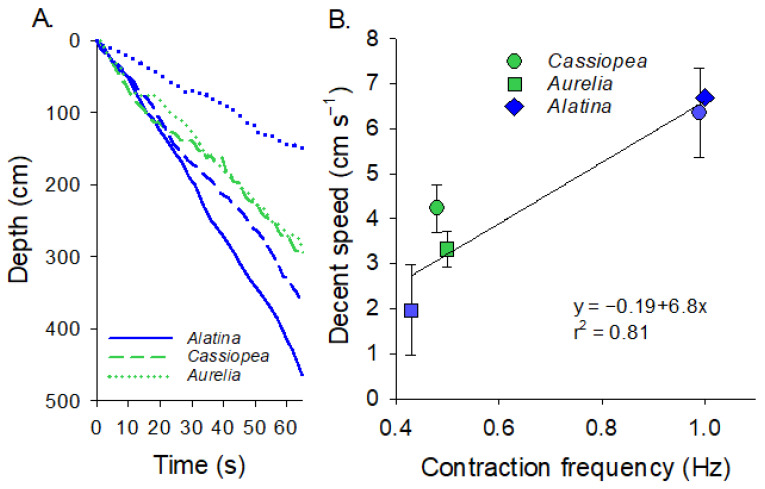
Swimming speeds of the different species. (**A**) Depth versus time for the different species. For *Cassiopea* spp. and *Aurelia aurita*, we had profiles for animals with their rhopalia intact (unexcised, blue) and with their rhopalia removed (excised, green). (**B**) The relationship between jellyfish bell contraction frequency and the speed at which the jellyfish descended. Circles are *Cassiopea*, squares are *Aurelia* and diamonds are *Alatina*. Blue are unexcised and green are excised.

The speed at which the medusae descended in the water column was directly related to the contraction rate (Figure 4A,B, Regression analysis, *p* < 0.0002). Medusan species did not seem to strongly vary in terms of their descent rate, with both *C. xamachana* and *A. alata* descending at the same rate when pulsing at the same frequency. However, *C. xamachana* did swim significantly faster than *A. aurita* when pulsing at 0.5 Hz (*T*-test, *p* < 0.05). A doubling of the contraction rate (0.5 vs. 1 Hz) almost doubled the descent speed of the medusae (3.7 vs. 6.5 cm s^−1^).

### 3.4. Contraction Frequency Limits

**Figure 5:**
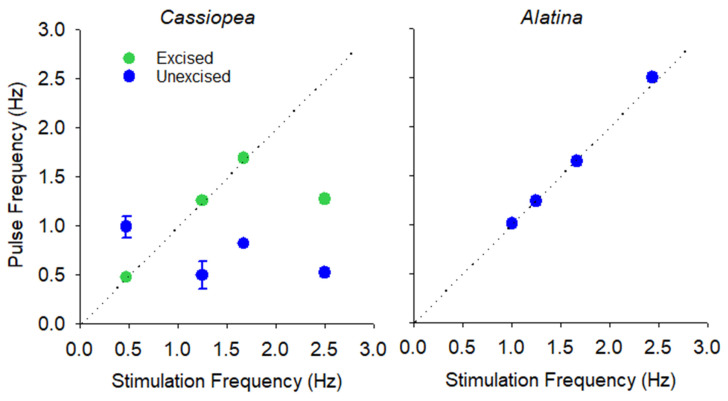
Relationship between stimulation frequency and jellyfish bell pulse frequency. Dotted line shows where the 1:1 relationship falls on the plot.

In the laboratory, we stimulated *C. andromeda* and *A. alata* at different rates to determine how rapidly each species could potentially contract their bells. We found a very high correlation between stimulus and contraction frequencies for excised *C. andromeda* (Figure 5) and non-excised *A. alata* (Figure 5). There was no correlation for non-excised *C. andromeda* (Figure 5). *A. alata* pulsed at rates as high as 2.5 Hz. However, *C. andromeda* was not able to contract its bell at rates greater than 1.67 Hz (Figure 5).

## 4. Discussion

Laboratory and field studies with the moon jellyfish, *Aurelia aurita*, demonstrate that scyphozoan swimming can be externally controlled using simple electrical impulses due to their relatively simple neuromuscular systems [13,15,16]. This feature makes jellyfish a promising platform for biohybrid robots capable of large-scale ocean sampling. However, *A. aurita* exhibits inherently low pulsation frequencies and slow swimming speeds, constraining applications such as deep vertical profiling. Moreover, inter-individual variability in responsiveness reduces reliability—some individuals fail to initiate swimming under stimulation, while others exhibit natural contractions that interfere with induced pulses [13]. These limitations restrict the operational versatility of jellyfish biohybrid robots.

To enhance the versatility of jellyfish biohybrid robots, we evaluated the ability of the swim controller to regulate swimming in multiple jellyfish species. Swimming in two distantly related medusae—the rhizostome *Cassiopea* spp. and the cubomedusa *Alatina alata*—was successfully controlled. Both taxa are stronger swimmers than *Aurelia aurita*, enabling faster vertical profiling. However, in *Cassiopea*, endogenous bell pulsations interfered with controller-driven pulses. Removal of the rhopalia, which initiate and coordinate natural contractions, eliminated this interference and rendered *Cassiopea* consistently responsive to the swim controller. In contrast, both *A. aurita* and *A. alata* remained responsive even with intact rhopalia. Thus, controller responsiveness is species- and potentially individual-specific, introducing uncertainty in performance. Nevertheless, rhopalia removal eliminates natural endogenous pulsations while preserving responsiveness, markedly improving control and reliability. Furthermore, the capacity to employ species with distinct swimming behaviors and physiological traits broadens the operational versatility of jellyfish biohybrid robots.

Jellyfish swimming is governed by rhopalia, pacemaker structures that generate electrical impulses to initiate bell contractions [20]. Typically, eight or more rhopalia are distributed around the bell margin; when one fires, it suppresses the others, ensuring a single pacemaker controls each pulse [20,21,22]. In contrast, impulses from the swim controller do not inhibit rhopalial activity, allowing natural signals to interfere with controller-driven pulses. This can disrupt swimming kinematics or, as observed in *Cassiopea* spp., render the controller ineffective. *Cassiopea* exhibited a higher intrinsic pulse frequency than *Aurelia aurita* and exceeded the stimulation frequency of the controller. Previous studies have shown that in some medusae, the fastest pacemaker dominates swimming activity [20,21,22]. Consequently, with intact rhopalia, *Cassiopea*’s endogenous pacemakers overrode the controller. Rhopalia removal eliminated pacemaker activity and endogenous contractions, leaving the swim controller as the sole driver of bell pulsations. Under these conditions, contractions occurred reliably, independent of the jellyfish’s natural pacemaker frequency.

Demonstrating that different jellyfish taxa are responsive to the swim controller increases the versatility of jellyfish biohybrid robots. The pacemaker system that controls swimming is similar among all scyphozoan jellyfish, such as *A. aurita* and *Cassiopea* spp. Likewise, cubomedusae, or box-jellyfish, also share a very similar pacemaker system [20]. Therefore, we expect that most, if not all, scyphozoan and cubozoan jellyfish will respond well to removal of their rhopalia and to the swim controller. However, the neuromuscular organization of hydromedusae is fundamentally different [20]. The pacemakers in hydromedusae are not localized to rhopalia, rather, they are integrated directly into a marginal nerve ring that encircles the bell [23,24]. In fact, the entire bell margin of hydromedusae needs to be removed to stop their swim pulsations [20]. Additionally, hydromedusae are much smaller than most scyphomedusae and cubomedusae, limiting the size of the payload that they can transport and their utility as biohybrid robots. Nevertheless, the taxonomic diversity of scyphozoans and cubozoans provides substantial opportunities for tailoring jellyfish biohybrid robots to distinct applications.

Medusan species exhibit considerable variation in size, swimming behavior, and physiology. Across the taxa examined, pulsation rate emerged as the primary determinant of swimming speed. For instance, *Cassiopea* and *A. alata* achieved similar speeds when pulsing at equivalent frequencies, consistent with their classification as fast-swimming rhizostomes and cubomedusae. Doubling *Cassiopea*’s pulsation rate nearly doubled its swimming speed; however, even at identical frequencies, *A. aurita* swam significantly slower (*t*-test, *p* < 0.05). Further, not all species are capable of pulsing at high pulsation rates. We found that while *A. alata* was capable of pulsing reliably at 2.5 Hz, *Cassiopea* was not able to pulse faster than 1.7 Hz. This is due to the fact that bell relaxation is a passive process controlled by the elastic properties and shape of the medusan bell rather than muscles. Therefore, the time it takes the bell to relax will limit the fastest pulse frequency. Cubomedusae, like *A. alata*, are jet-propelled—rather than rowing-propelled like the scyphomedusae—and jetting jellyfish are generally capable of faster bell kinematics. These species-specific biomechanical traits may be influential variables when selecting jellyfish for biohybrid robotic applications.

Additional species-specific traits, such as body size and swimming efficiency, may further expand the utility of jellyfish biohybrid robots. *A. aurita* has been reported to be the most efficient swimmer among both aquatic and terrestrial animals [25], and although all scyphozoans exhibit high efficiency via rowing propulsion, *A. aurita* is nearly twice as efficient as the rhizostome *Stomolophus meleagris*. Whether this efficiency translates to swim-controller-driven propulsion remains unknown, and future work is needed to quantify battery consumption relative to swimming efficiency. Size also varies substantially among medusae: while the species tested here were <20 cm in diameter, the rhizostome *Rhizostoma pulmo* can exceed 100 cm. Increased bell size directly increases potential payload capacity, including the swim controller and additional sensors. Substantial additional research will be necessary to fully evaluate the potential versatility of diverse jellyfish as biohybrid robots.

## 5. Conclusions

Our findings show that multiple different types of jellyfish can be developed as biohybrid robots and that reliability can be improved when endogenous pulses are eliminated by the removal of rhopalia. But performance depends on species-specific traits such as swimming mechanics, efficiency, and body size. These factors influence reliability, speed, and payload capacity, making species choice critical for optimizing function. Together, these findings highlight the importance of species selection in optimizing jellyfish biohybrid robots for distinct oceanographic applications, while also underscoring the need for further investigation into efficiency, control reliability, and scalability.

## Data Availability

Data from this project can be downloaded from https://submit.bco-dmo.org/project/r0LV5WK8wqzu1wQ7 (accessed 17 October 2025).

## Supplementary Materials

The following supporting information can be downloaded at: https://www.mdpi.com/article/10.3390/biomimetics10120810/s1, Figure S1: Images of swimming *Aurelia aurita* and *Cassiopea xamachana* with and without intact rhopalia. Rhopalia are tiny structures located around the bell margin. Small sections of the bell that included the rhopalia were cut out to excise the rhopalia.; Figure S2: Schematic of experimental set-up. To measure pulse frequency in the laboratory, we video-recorded side-illuminated medusae using a GoPro camera suspended from above.; Figure S3: Time series of individual *Aurelia aurita* swimming in the water column offshore from Long Key, Florida. These are 4 different individuals, 2 non-excised (with rhopalia; blue lines) and 2 excised (green lines). Orange lines indicate times when the jellyfish were stimulated to swim.; Figure S4: Time series of individual *Cassiopea xamachana* swimming in the water column offshore from Long Key, Florida. These are 4 different individuals, 2 non-excised (with rhopalia; blue lines) and 2 excised (green lines). Orange lines indicate times when the jellyfish were stimulated to swim.; Figure S5: Time series of individual *Alatina alata* swimming in the water column offshore from Kona, Hawaii. These are 2 different individuals; both were non-excised (with rhopalia; blue lines). Orange lines indicate times when the jellyfish were stimulated to swim.
