# Supplementary material for: Increasing the Reliability and Versatility of Jellyfish Biohybrid Vehicles via Species Selection and Rhopalia Removal
Source: Biomimetics (Basel). 2025 Dec 3;10(12):810. doi: 10.3390/biomimetics10120810 (PMC12730998; doi:10.3390/biomimetics10120810)
Supplement: Supplementary file 1 [file biomimetics-10-00810-s001.zip › biomimetics-3961996-supplementary.pdf]

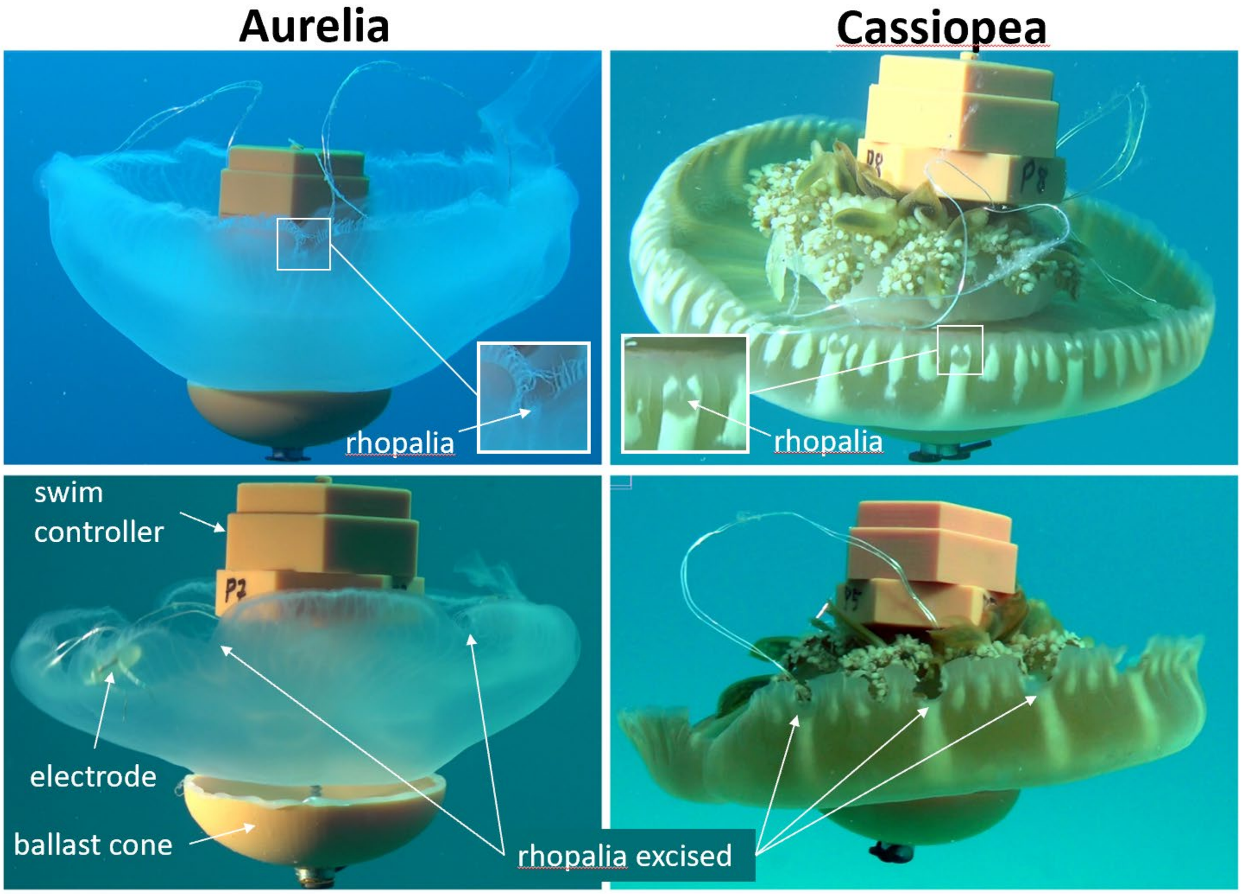

Supplementary fig. S1. Images of swimming *Aurelia aurita* and *Cassiopea xamachana* with and without intact rhopalia. Rhopalia are tiny structures located around the bell margin. Small sections of the bell that included the rhopalia were cut out to excise the rhopalia.

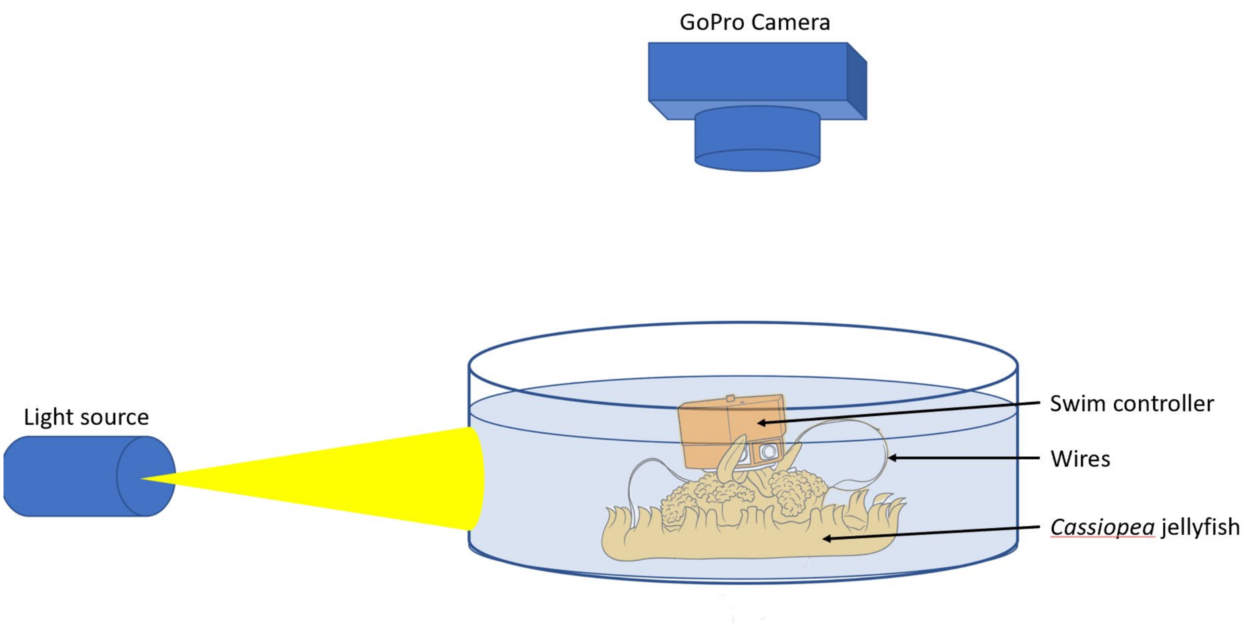

Supplementary fig. S2. Schematic of experimental set-up. To measure pulse frequency in the laboratory we video recorded side-illuminated medusae using a GoPro camera suspended from above.

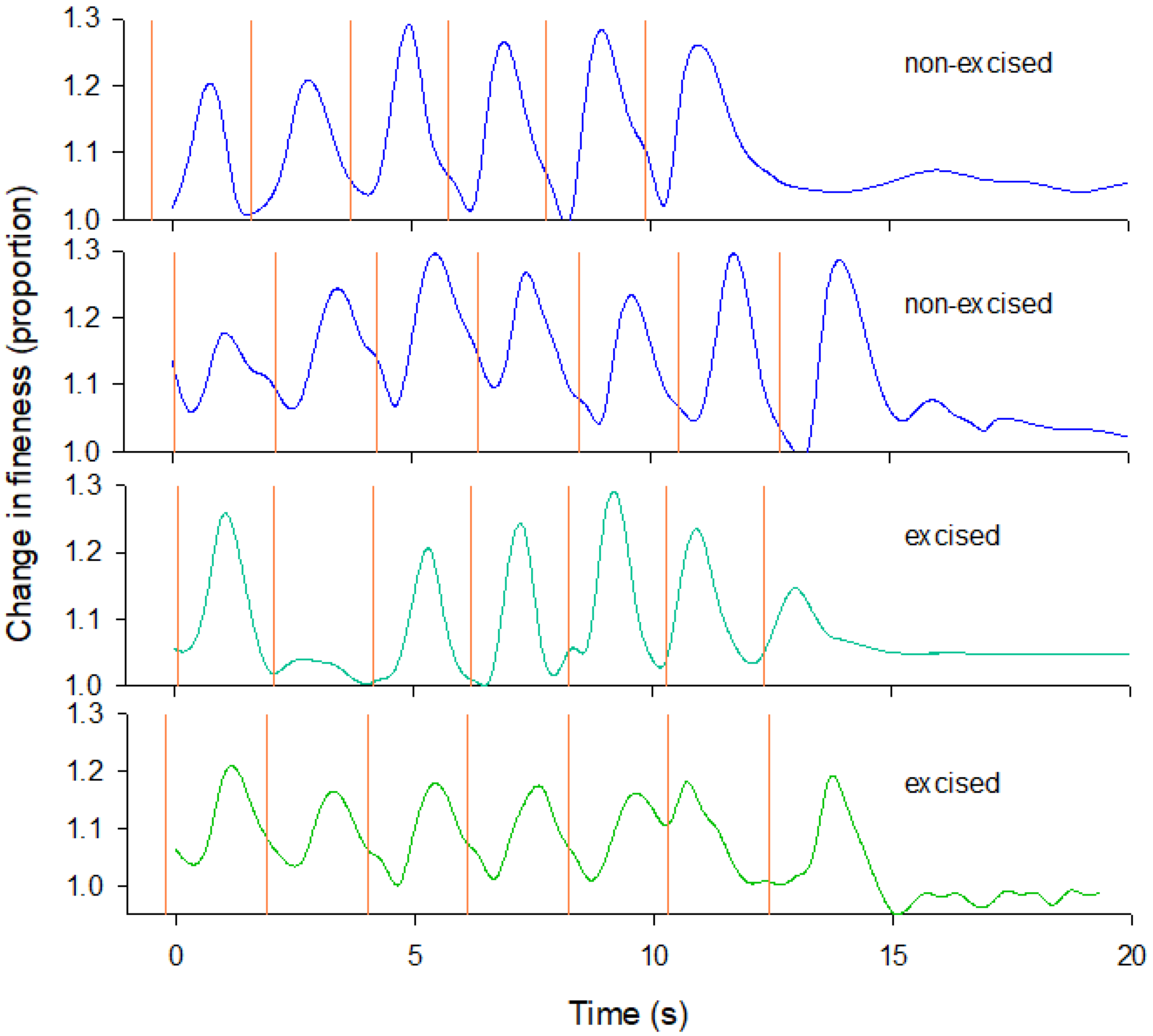

Supplementary Fig. S3. Time series of individual *Aurelia aurita* swimming in the water column offshore from Long Key, Florida. These are 4 different individuals, 2 non-excised (with rhopalia; blue lines) and 2 excised (green lines). Orange lines indicate time when they jellyfish were stimulated to swim.

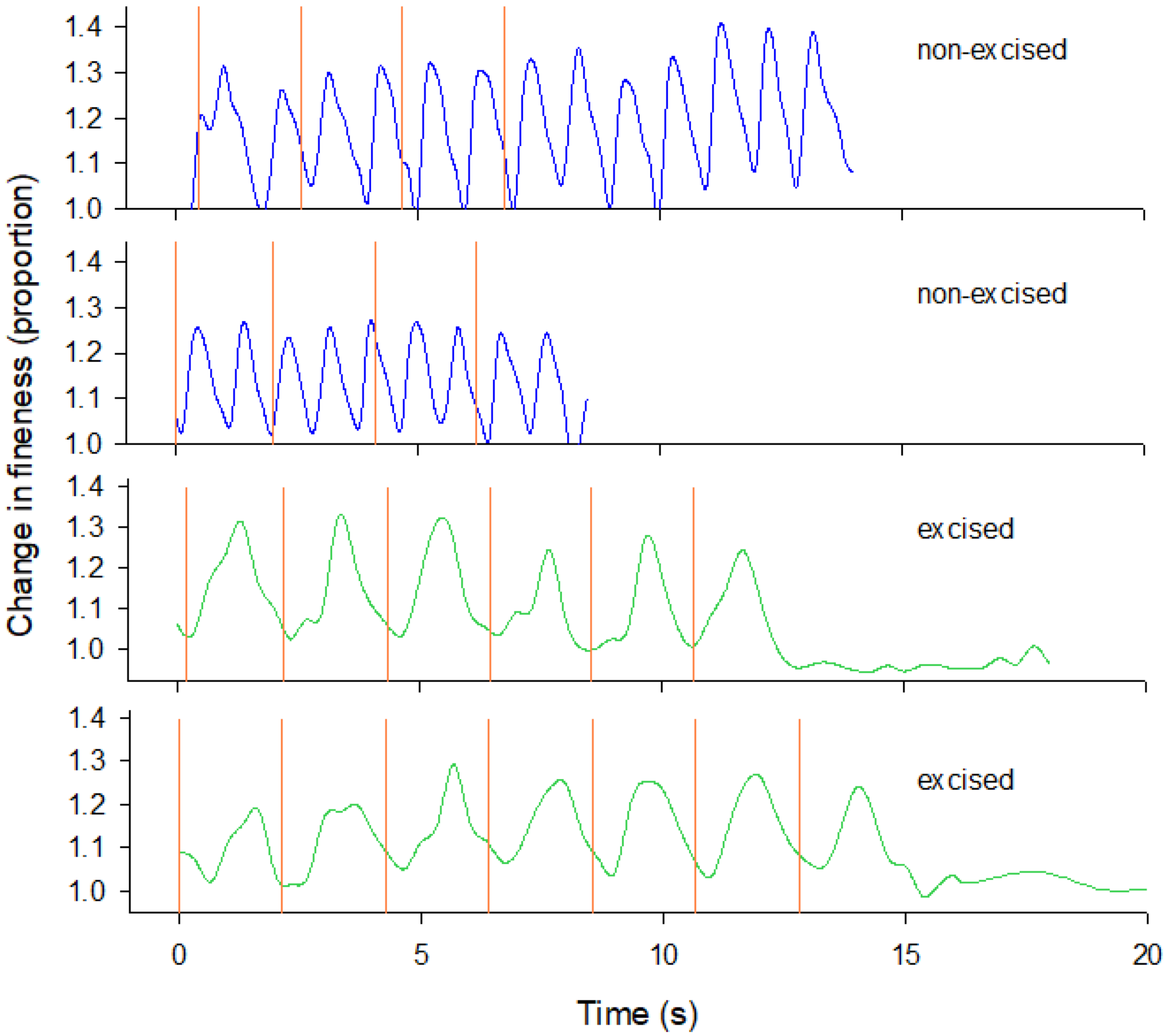

Supplementary Fig. S4. Time series of individual *Cassiopea xamachana* swimming in the water column offshore from Long Key, Florida. These are 4 different individuals, 2 non-excised (with rhopalia; blue lines) and 2 excised (green lines). Orange lines indicate time when they jellyfish were stimulated to swim.

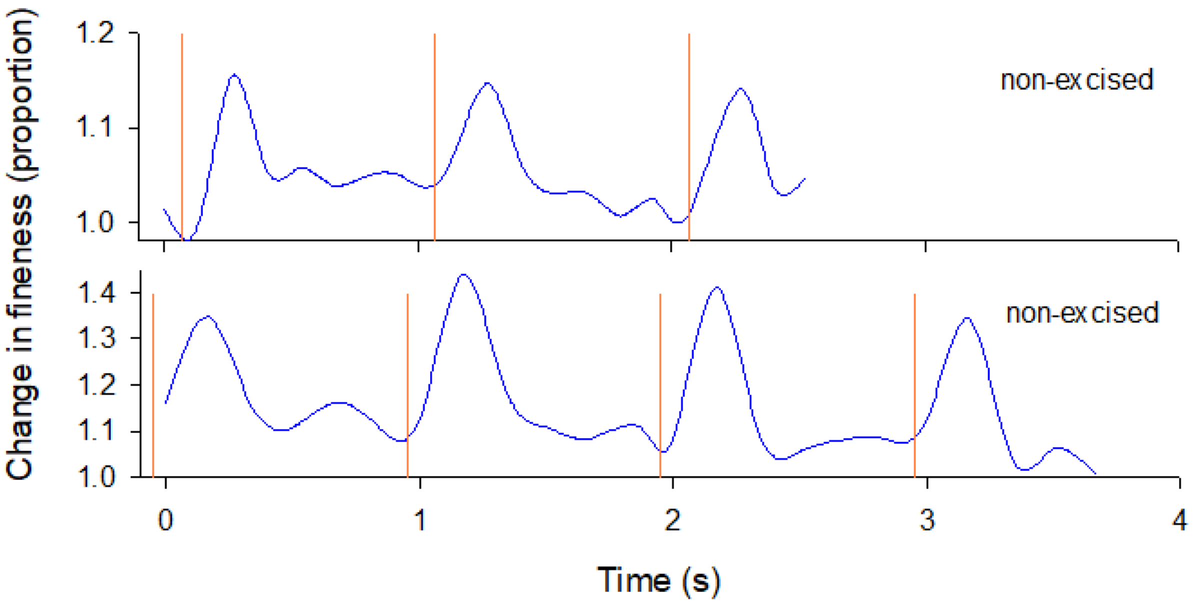

Supplementary Fig. S5. Time series of individual *Alatina alata* swimming in the water column offshore from Kona, Hawaii. These are 2 different individuals both were non-excised (with rhopalia; blue lines). Orange lines indicate time when they jellyfish were stimulated to swim.
